# Role of HIV-1 subtype C envelope V3 to V5 regions in viral entry, coreceptor utilization and replication efficiency in primary T-lymphocytes and monocyte-derived macrophages

**DOI:** 10.1186/1743-422X-4-126

**Published:** 2007-11-24

**Authors:** Vasudha Sundaravaradan, Suman R Das, Rajesh Ramakrishnan, Shobha Sehgal, Sarla Gopalan, Nafees Ahmad, Shahid Jameel

**Affiliations:** 1 Department of Immunobiology, College of Medicine, University of Arizona, Tucson, AZ 85724, USA; 2 Virology Group, International Center for Genetic Engineering and Biotechnology, New Delhi, India; 3 Departments of Pathology and Obstetrics and Gynecology, Post Graduate Institute of Medical Education and Research, Chandigarh, India; 4 NIAID, National Institutes of Health, Bethesda, MD 20892, USA; 5 Department of Molecular Virology & Microbiology, Baylor College of Medicine, Houston, TX 77030, USA

## Abstract

**Background:**

Several subtypes of HIV-1 circulate in infected people worldwide, including subtype B in the United States and subtype C in Africa and India. To understand the biological properties of HIV-1 subtype C, including cellular tropism, virus entry, replication efficiency and cytopathic effects, we reciprocally inserted our previously characterized envelope V3–V5 regions derived from 9 subtype C infected patients from India into a subtype B molecular clone, pNL4-3. Equal amounts of the chimeric viruses were used to infect T-lymphocyte cell lines (A3.01 and MT-2), coreceptor cell lines (U373-MAGI-CCR5/CXCR4), primary blood T-lymphocytes (PBL) and monocyte-derived macrophages (MDM).

**Results:**

We found that subtype C envelope V3–V5 region chimeras failed to replicate in T-lymphocyte cell lines but replicated in PBL and MDM. In addition, these chimeras were able to infect U373MAGI-CD4^+^-CCR5^+ ^but not U373MAGI-CD4^+^-CXCR4^+ ^cell line, suggesting CCR5 coreceptor utilization and R5 phenotypes. These subtype C chimeras were unable to induce syncytia in MT-2 cells, indicative of non-syncytium inducing (NSI) phenotypes. More importantly, the subtype C envelope chimeras replicated at higher levels in PBL and MDM compared with subtype B chimeras and isolates. Furthermore, the higher levels subtype C chimeras replication in PBL and MDM correlated with increased virus entry in U373MAGI-CD4^+^-CCR5^+^.

**Conclusion:**

Taken together, these results suggest that the envelope V3 to V5 regions of subtype C contributed to higher levels of HIV-1 replication compared with subtype B chimeras, which may contribute to higher viral loads and faster disease progression in subtype C infected individuals than other subtypes as well as rapid HIV-1 subtype C spread in India.

## Introduction

The steepest increase in new cases of human immunodeficiency virus type 1 (HIV-1) infection has taken place in South America [1] and South/Southeast Asia [2], of which India is experiencing a rapid and extensive spread of infection. National surveys in India have shown that the spread in India is primarily heterosexual in the metropolitan and coastal cities, and via intravenous drug use in the Northeast bordering Myanmar [3,4]. The HIV-1 sequences analyzed from different cohorts from several regions in India suggests that HIV-1 subtype C is the predominant subtype found in India [3-8]. It has also been shown in several African and South American studies that subtype C rapidly predominates over all the other HIV-1 subtypes after being introduced in those populations [1,9], suggesting that subtype C may soon become the prevalent subtype worldwide. However, the biological properties of HIV-1 subtype C viruses that may influence its rapid spread are not known.

HIV-1 envelope gp120 interacts with CD4 receptor and CXCR4 or CCR5 coreceptor [10-13] on T lymphocytes, monocytes/macrophages and other cell types [11,14,15] to enter target cells. Analysis of *env *gp120 sequences from a large number of HIV-1 isolates shows that gp120 is made up of five variable regions (V1 to V5) that are interspersed with conserved regions [16]. The potential pathogenic region of HIV-1 presumably lies within these variable regions, especially in the V3 region comprising of 35 amino acids arranged in a disulphide loop involving two cysteines [10]. The hypervariable region 3, the V3 region, is functionally important in virus infectivity [17-19], virus neutralization [13,20-22], replication efficiency and host cell tropism [10,23], whereas the V1–V2 regions influence replication efficiency in macrophages by affecting virus spread [24,25]. In addition, the variable loops V4 and V5 of gp120 are less flexible regions of the proteins and may play roles in CD4 binding and neutralizing antibody responses [26,27]. The mechanisms by which the V3 domain and other regions of the *env *glycoprotein control cell tropism were described by identifying two distinct co-receptors, fusin (CXR4) and CCR5, for the entry of T-lymphotropic and macrophage-tropic HIV-1, respectively [11,15]. The region responsible for determining coreceptor utilization was examined by Choe et al., [12] and showed that the V3 region was responsible for interacting with this co-receptor. Several studies have shown that a reciprocal transfer of an HIV-1 R5 clones' V3 region into an X4 molecular clone changed its tropism to allow infection and replication in macrophages [10,19,23,28-30]. However, most of the data on viral infectivity, coreceptor utilization, replication efficiency and cytopathic effects have been obtained from HIV-1 subtype B, and very limited information is available on subtype C viruses, especially those from India.

In this study, we have characterized the biological properties of HIV-1 subtype C envelope V3 to V5 regions by constructing chimeric recombinant viruses containing subtype C envelope V3–V5 regions from nine infected patients from India [5] into subtype B infectious molecular clone, pNL4-3. We show that the envelope V3–V5 regions of HIV-1 subtype C changed the tropism of HIV-1 NL4-3 from X4 to R5 and contributed to the increased virus entry and replication efficiency in primary blood T-lymphocytes (PBL) and monocyte-derived macrophages (MDM) compared with subtype B viruses. This higher replication efficiency of subtype C compared with subtype B may contribute to a higher viral load and faster disease progression in patients infected with HIV-1 subtype C viruses in the Indian population.

## Results

### Characterization and comparison of subtype C envelope V3–V5 chimeras' sequences with known isolates

**Figure 1:**
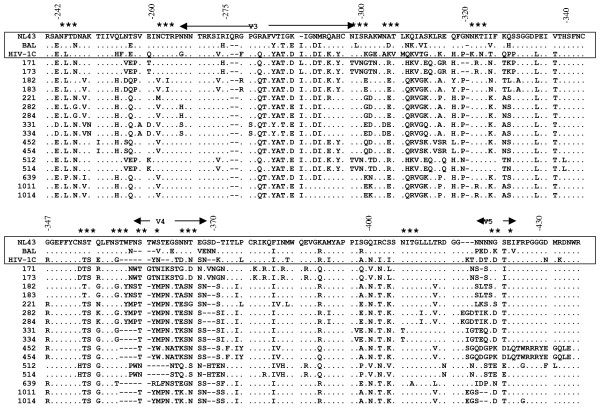
Comparison of envelope (V3 to V5 regions) from subtype C chimeras with subtype B (X4 and R5) and C envelope sequences. The sequences of subtype C *env *chimeras used in this study were analyzed by performing multiple sequence alignment with parental clone HIV-1_NL4-3 _as a reference and HIV-1_BaL _and known HIV-1 subtype C envelope regions for comparison. Subtype C chimeras are designated by numbers. Dots indicate a match with the reference sequence whereas substitutions are indicated by the single letter code for the changed amino acid. Gaps are shown as dashes. Structural elements of the envelope are indicated by spanning arrowheads and glycosylation sites are indicated by asterisk. Amino acid positions are indicated to denote the amino acid numbers of the complete envelope gp120.

We confirmed the reciprocal insertion of the sixteen envelope V3–V5 region sequences (Fig. 1) from nine subtype C infected patients' isolates from India (Table 1) that were sequenced before [5] into pNL4-3 by nucleotide sequencing. Two clones were selected from each patient for reciprocal insertion. The patients harbored various stages of HIV disease (I to IVE) based on the 1987 CDC classification. We also compared the subtype C *env *V3 to V5 regions chimeras with HIV-1_NL4-3_, HIV-1_BaL _and a known *env *V3–V5 region sequence of subtype C (Fig. 1). All the subtype C *env *V3–V5 clones showed a greater similarity to the R5 virus HIV-1_BaL _than HIV-1_NL4-3_. The amino acids critical for the R5 phenotype, including the Y at position 283 and E or D at position 287 were present in all subtype C clones. The amino acid H at position 275 seen in HIV-1_Ba-L _which is also important for determining R5 tropism was present only in the clones from patient 17 (clones 171 and 173). The amino acid sequences between positions 310–315 and 350–373 were significantly different from HIV-1_NL4-3 _clone but were very similar to the previously known sequence of subtype C envelope region. It is interesting to note that a critical glycosylation site, which includes the first cysteine of the V3 loop, was mutated in all the clones except those obtained from patient 17 and 5 (clones 171, 173, 512, 514). The sequence analysis of all the clones indicate that these clones are different from HIV-1_NL4-3 _envelope sequences but very similar to envelope sequences from known subtype C clones and to R5 tropic viruses such as HIV-1_BaL_.

**Table 1:** Patient demographics, possible source and risk factor of transmission and CDC disease classification.

Patient Code	Chimeras Number	Age/Sex	Possible source	Risk factor	CDC classification
AP.17	171, 173	27/M	Africa/Germany	Promiscuous heterosexual	IV-C, D
AP.18	182, 183	14/M	Punjab (India)	Blood transfusion	IV-E
AP.2	221A	20/M	Dubai/Punjab	Promiscuous heterosexual	II
AP.28	282, 284	28/M	Kolkata (India)	Truck driver	IV
AP.33	331, 334	26/M	Punjab	Hemophiliac	I
AP.4	452, 454	25/M	Mumbai (India)	Promiscuous heterosexual	IV-C
AP.5	512, 514	23/F	Africa/Germany	Spouse with AIDS	II
AP.6	639	27/M	Punjab	Promiscuous heterosexual	II
AP.10	1011, 1014	25/M	Mumbai	Blood transfusion	IV-C

CDC-Center for Disease Control and Prevention

### Computational prediction of coreceptor usage using V3 amino acid sequence of subtype C chimeras

We also used the Position Specific Scoring Matrix (PSSM) bioinformatics tool (X4/R5) to predict coreceptor usage by the Subtype C patient *env *V3–V5 region sequences [31] and calculated scores for each clone are shown in Table 2. A score of -6.96 was used as a cut off for R5 strains and a score of -2.88 was used as a cutoff for X4 strains. Most of these scores were higher than -6.96 especially AP.17 sequence which showed a score of -3.58 clearly below the cut-off score for X4 strains. Thus, all the clones were clearly predicted to be R5 tropic. We also used the PSSM (sinsi) matrix to analyze these sequences and these scores were in the range of -7.5 to -11.53. This predicts high R5 tropism and a NSI phenotype for all the Indian isolates examined. Higher numbers of positively charged amino acids (R/K/H) have been correlated with the likelihood of CXCR4 use. In all of the Indian sequences in this study, lower numbers of positive charges, in the range of 5 to 7, were found (Table 2). Thus, in an in-silico prediction model, all of the V3–V5 sequences of HIV-1 Indian isolates were predicted to show R5-tropism.

**Table 2:** Prediction of coreceptor usage by Position Specific Scoring Matrix (PSSM) bioinformatics tool that predicts coreceptor usage.

Patient/Clone	Software	Score	Pred	X4 pct	R5 pct	Geno	Pos chg	Net Chg	Percent
AP.17/171, 173	X4/R5	-3.58	0	0.47	0.98	SE	6	4	0.83
	Sinsi	-7.05	0	0.04	0.90	SE	6	4	0.83
AP.18/182, 183	X4/R5	-6.48	0	0.27	0.95	SD	6	4	0.70
	Sinsi	-10.73	0	0.04	0.58	SD	6	4	0.70
AP.2/221A	X4/R5	-7.83	0	0.22	0.9	SD	7	5	0.57
	Sinsi	-9.5	0	0.04	0.73	SD	7	5	0.57
AP.28/282, 284	X4/R5	-8.89	0	0.22	0.88	SE	7	5	0.44
	Sinsi	-12.12	0	0.01	0.2	SE	7	5	0.44
AP.33/331, 334	X4/R5	-7.36	0	0.24	0.92	SE	6	4	0.62
	Sinsi	-10.61	0	0.04	0.51	SE	6	4	0.62
AP.4/452, 454	X4/R5	-6.50	0	0.27	0.95	SD	5	2	0.67
	Sinsi	-11.53	0	0.01	0.32	SD	5	2	0.67
AP.5/512, 514	X4/R5	-9.74	0	0.22	0.82	SE	5	3	0.33
	Sinsi	-10.69	0	0.01	0.50	SE	5	3	0.33
AP.6/639	X4/R5	-9.06	0	0.22	0.86	SD	6	4	0.48
	Sinsi	-12.26	0	0.01	0.17	SD	6	4	0.48
AP.10/1011, 1014	X4/R5	-9.36	0	0.22	0.83	SE	6	4	0.31
	Sinsi	-11.16	0	0.01	0.04	SE	6	4	0.31

### Replication of subtype C chimeric viruses in T-Lymphocyte cell lines

**Figure 2:**
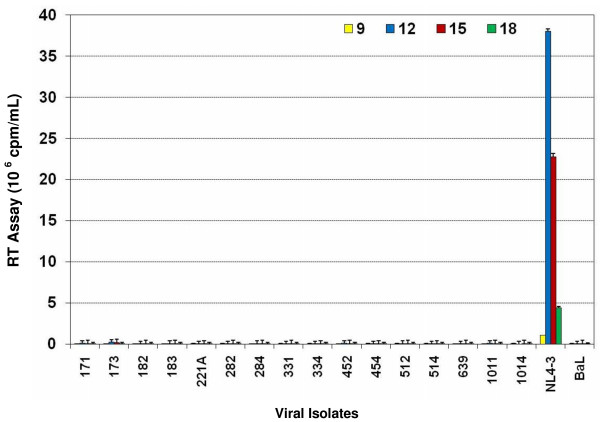
Replication of HIV-1 subtype C *env *V3–V5 region chimeras in T-lymphocyte (A3.01) cell line. A3.01 cells (1 × 10^6 ^cells/well) were infected with equal amounts (RT counts) subtype C *env *chimeras (171 to 1014), parental HIV-1_NL4-3 _and HIV-1_BaL_. Virus production was measured by reverse transcriptase (RT) assay in culture media harvested every 3 days and the cells fed with appropriate media. The results are presented as cpm/ml ± SD of five separate triplicate experiments. The subtype C chimeras were unable to replicate in A3.01 cell line.

We first sought to determine whether the subtype C *env *chimeras retained the lymphotropic properties of the parental clone HIV-1_NL4-3 _by infecting the T cell line A3.01 with the subtype C chimeric viruses and parental virus, HIV-1_NL4-3_. The T-cell line A3.01 expresses CD4 and CXCR4 but no CCR5. Our results showed that the parental X4 tropic HIV-1_NL4-3 _productively infected and replicated in A3.01 over a 27-day infection period. However, the subtype C chimeras did not replicate in A3.01 cell line (Fig. 2), suggesting that these chimeras were no longer T-cell line tropic, unlike the parental clone. These results further demonstrated that replacement of the V3–V5 regions of envelope of HIV-1_NL4-3 _with subtype C envelope from infected patients was responsible for the lack of capacity to use CXCR4 as a coreceptor for these chimeric viruses. These data also suggest that the integrated proviruses that were present in these subtype C infected patient samples were not X4 tropic.

### Coreceptor utilization of chimeric subtype C viruses

We then determined the coreceptor utilization of the subtype C *env *chimeras (clone designation in Fig. 1 and Table 1) using U373-MAGI indicator cell lines. These cell lines express the CD4 receptor in conjunction with either CCR5 or CXCR4 as a coreceptor and can be infected with either macrophage tropic (R5) or lymphotropic (X4) viruses, respectively. These cells contain an inducible β-galactosidase reporter driven by HIV-1 LTR that can be used as an indicator for entry. The known SI isolate HIV-1_NL4-3_, NSI isolate HIV-1_BaL_, and primary isolates were used as controls. As shown in Table 3, all the subtype C *env *chimeras were able to infect the U373-MAGI-CCR5 cell line but were unable to infect the U373-MAGI-CXCR4 cell line. This data also correlates well with the insilico prediction models and confirms the coreceptor usage of these viruses. This data suggests that the cloning of these chimeras yielded functional envelope regions that can bind CD4/CCR5 and allow virus entry and production of early viral genes. As seen by the counts for infectivity of the MAGI-CCR5 line, the chimeras showed considerable differences in infectivity. Some chimeras (171, 173) demonstrated remarkably high levels of infectivity when compared to other chimeras and primary isolate controls indicating an increased rate of entry for these chimeras. The levels of entry using increasing virus counts (5000 and 10,000 RT counts) during infection showed increased level of entry for the same chimeras. This shows that the envelope region of subtype C HIV-1 obtained from the patient samples shows an R5 phenotype for the virus infecting the patient. These results also suggest that all the chimeric viruses obtained could have had different rates of entry when infecting target cells. This can be attributed to the differences in the V3 region of the subtype C chimeric DNA, reflecting the differences found in patient samples. This also adds to previous work done by others [4,32,33] showing that the coreceptor utilization of subtype C HIV-1 could be predominantly R5 even late in infection.

**Table 3:** Coreceptor usage by HIV-1 subtype C chimeras in U373-MAGI-CCR5 and U373-MAGI-CXCR4 cell lines.

	**MAGI-CCR5**		**MAGI-CXCR4**		
**Infection counts →**	**5000**	**10000**	**5000**	**10000**	**Phenotype from MT-2**

**CHIMERA_**	**No. of blue cells**		**No. of blue cells**		

171	21	99	-	-	NSI
173	23	92	-	-	NSI
182	9	18	-	-	NSI
183	7	12	-	-	NSI
221A	0	2	-	-	NSI
282	9	30	-	-	NSI
284	2	6	-	-	NSI
331	13	31	-	-	NSI
334	1	2	-	-	NSI
452	23	65	-	-	NSI
512	1	3	-	-	NSI
514	1	4	-	-	NSI
639	1	2	-	-	NSI
1011	11	26	-	-	NSI
1014	16	33	-	-	NSI
2099 (B-R5)	8	19	-	-	NSI
2101 (B-X4/R5)	5	10	10	21	SI
3041 (C-R5)	7	34	-	-	NSI
5441 (C-X4)	-	-	23	38	SI

NSI – non-syncytium inducing, SI – syncytium inducing, - indicate no entry due to lack of blue cells.

### Syncytium inducing capacity of subtype C chimeras

We examined the syncytium-inducing ability of the subtype C *env *chimeras by infecting MT-2 cell lines with the chimeric viruses. Viruses that produce a greater than four syncytia per field were denoted as syncytium inducing (SI) phenotype and the viruses that did not induce any syncytia were called as non-syncytium inducing (NSI) phenotype. As shown in the Table 3, all of the subtype C V3–V5 region chimeras failed to produce any syncytia in MT-2 cells and therefore are of the NSI phenotype (similar to known R5 isolates HIV-1_BaL_). The control parental virus HIV-1_NL4-3 _that has a known SI phenotype produced significant levels of syncytia (at least 10 per field of view). As expected, the R5 viruses used as control also did not produce syncytia in culture. These data suggest that the envelope sequences from subtype C infected patient samples render them less cytopathic as compared to HIV-1_NL4-3_. We also confirmed virus production in MT-2 cells and the syncytium formation correlated to virus production in our partial *env *(V3–V5) region chimeras (NSI) and R5 isolates as measured by RT assay in the culture medium (not shown).

### Replication of subtype C chimeras in primary peripheral blood T-lymphocytes

**Figure 3:**
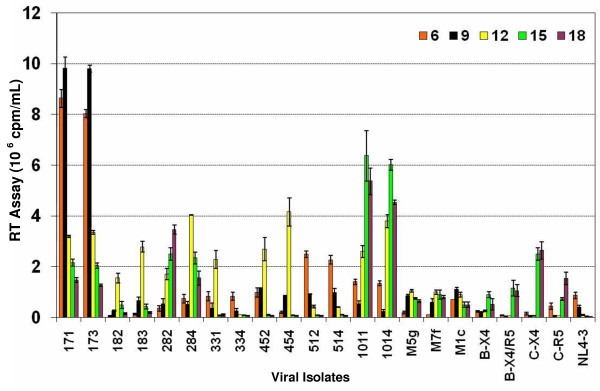
Replication of HIV-1 subtype C *env *V3–V5 region chimeras in primary peripheral blood T-lymphocytes (PBL). PBL (1 × 10^6 ^cells/well) were stimulated with PHA and infected with equal amounts (reverse transcriptase counts) of subtype C *env *V3–V5 region chimeras, subtype B *env *V3 region chimeras, primary subtype B isolates, primary subtype C isolates and parental HIV-1_NL4-3_. Cells were fed every 3 days with appropriate medium and virus production was measured in the culture supernatant by RT assay. The data are presented as cpm/ml ± SD on triplicate experiments and are based on PBL from five different donors.

Primary T-lymphocytes from peripheral blood express CD4 and both chemokine receptors, CXCR4 and CCR5. Since PBL express both CCR5 and CXCR4, they are capable of supporting the replication of both R5 and X4 tropic viruses. All the subtype C *env *chimeras, which did not replicate in A3.01, were able to replicate in PBL showing that there was no inherent defect in their replication as a results of the reciprocal insertion of the V3–V5 region from patient samples (Fig. 3). Although we have demonstrated the entry of these chimeric viruses using the MAGI cell experiments, the replication kinetics shown in Figure 3 confirm the capacity of these viruses to enter and replicate well in culture.

The replication kinetics of subtype C chimeras in PBL showed that these chimeras replicated at a higher efficiency as compared to subtype B chimeras (M5g, M7f, and M1c [34]) and subtype B isolates. Close observation showed that chimeras 171 and 173 replicated and peaked much earlier in infection as compared to the other chimeras. The V3–V5 region of these chimeras came from a patient who demonstrated advanced disease (Table 1) [5]. Although some chimeras (284 and 331) peaked relatively late in infection, they peaked at higher levels than the subtype B chimeras. All the subtype C chimeras also replicated at levels higher than the subtype B primary isolates. The replication data of the subtype C chimeras (Fig. 3) correlated well with the rate of entry seen in the MAGI cell line experiments (Table 3). The chimeras that scored higher numbers in MAGI cell experiments (Table 3) peaked earlier in viral infection experiments (Fig. 3). Comparative rates of entry of chimeras correspond with the peak of viral replication, where 171 and 173 with highest level of entry in MAGI cells showed very early and high peaks and 284 and 331 chimeras with much lower level of entry showed lower and/or more delayed peaks. It is interesting to note that chimeras 171, 173, 512 and 514, which retained the first proximal glycosylation site of the V3 region (Fig 1), peaked very early (Day 6–9) during replication (Fig 3). Comparison of the primary isolates of subtype B and subtype C also showed that the subtype C primary isolates replicated better than the subtype B primary isolates. These data suggest that the envelope V3 to V5 regions of the subtype C influenced the rate of replication of HIV-1 in primary T-lymphocytes and determined the cellular tropism. Furthermore, there was a direct relationship between higher viral replication (Fig. 3) and advanced disease status of the patients (Table 1).

### Replication of subtype C chimeras in primary monocyte-derived macrophages

**Figure 4:**
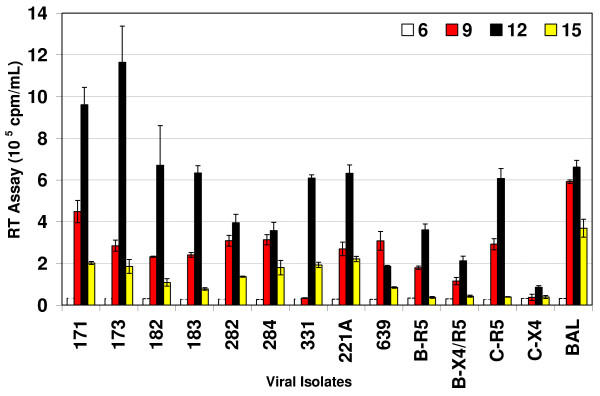
Replication of HIV-1 subtype C *env *V3–V5 region chimeras in primary blood monocyte-derived macrophages (MDM). MDM (0.5 × 10^6 ^cells/well) were infected with equal amounts (RT counts) of subtype C *env *V3–V5 region chimeras, primary subtype B isolates, primary subtype C isolates and HIV-1_BaL_. Cells were fed every 3 days with appropriate medium and the virus production was measured in the culture supernatant by RT assay. The data are presented as cpm/ml ± SD on triplicate experiments and are based on MDM from five different donors.

While primary monocyte derived macrophages (MDM) express CD4 and CCR5 and a low level of CXCR4, they support productive infection of R5 but not X4 viruses. As the subtype C chimeras showed a R5 phenotype (Table 3), replication kinetics of these chimeras were evaluated in MDM. Figure 4 shows the replication kinetics of subtype C chimeras in comparison with primary subtype B and C controls. The data clearly demonstrated that subtype C chimeras replicated better than subtype B viruses. The rate of replication of the subtype C chimeras in MDM also correlated with the rate of entry of the chimeras in MAGI-CCR5 cell line, further supporting the hypothesis that the increase in the replication of these chimeras is due to increase in the rate of entry. Both subtype B (2099) and subtype C (3041) primary R5 isolates replicated in MDM and subtype B dual tropic (X4/R5) virus (2101) also showed adequate replication in MDM. In addition, comparison of subtype B and subtype C primary isolates also showed that the subtype C primary isolates replicated better than subtype B primary isolates. These data suggest that the V3 to V5 regions of subtype C influenced increased replication of HIV-1 in MDM.

## Discussion

We have provided evidence regarding the role of the HIV-1 envelope V3–V5 regions from subtype C infected patients from India in virus entry, coreceptor utilization and replication efficiency in primary T-lymphocytes and macrophages in comparison with those of subtype B viruses. Our data suggest that the reciprocal insertion of HIV-1 subtype C infected patients' envelope V3 to V5 regions [5] into subtype B molecular clone, contributed to utilization of CCR5 coreceptor (Table 3) as well as higher levels of HIV-1 entry and replication efficiencies in primary T-lymphocytes (Fig. 3) and MDM (Fig. 4). The higher viral replication efficiencies of R5 phenotype of the chimeras correlated with advanced disease status of the patients (Table 1). Taken together, the increased replication capabilities of HIV-1 subtype C in T-lymphocytes and MDM may contribute to a high viral load, rapid disease progression, and spread in infected individuals [35,36].

We have demonstrated that subtype C envelope V3–V5 region chimeras showed increased levels of virus entry that correlated with an increased rate of replication in primary T-lymphocytes and MDM compared with subtype B chimeras and subtype B primary isolates. Careful observation indicates that chimeras with higher rate of entry peaked earlier during infection in primary cells. Unlike the lymphotropic (X4) parental clone HIV-1_NL4-3_, the subtype C *env *V3–V5 region chimeras were unable to replicate in T lymphocyte cell lines A3.01 (Fig. 2) and MT-2 (Table 3), suggesting that the chimeras had lost the T-cell line tropism of the parent clone NL4-3 because of reciprocal insertion of the V3–V5 region from subtype C patient samples. In addition, all of the subtype C *env *chimeras failed to produce any syncytia in MT-2 cells (Table 3), denoting NSI phenotypes, similar to the R5 but not NL4-3 isolates. Infection of U373-Magi-X4 and U373-Magi-R5 cell lines indicate that all our chimeric viruses and the control R5-tropic isolate HIV-1_BaL_, utilized the CCR5 coreceptor, whereas the parental HIV-1_NL4-3 _utilized the CXCR4 coreceptor (Table 3). These results are consistent with earlier reports that showed reciprocal insertion of the V3 region of an R5 isolate into an X4 molecular clone altered the tropism of an X4 isolate to an R5 phenotype [10,19,23,28-30].

Our in-silico analysis of the V3 sequences of the subtype C isolates from India predicted R5 tropism (Table 2). This also explains why these viruses replicated in primary blood Tlymphocytes (Fig. 3) but failed to replicate in the T4 lymphocyte cell lines, A3.01 (Fig. 2) and MT-2 because primary T-lymphocytes express CCR5, whereas and A3.01 and MT-2 do not. This data further supports the predominance of R5 phenotype in subtype C infected patients [37,38] and its maintenance during symptomatic AIDS. Several of the patients that exhibited advanced stages of HIV disease (Table 1) also harbored R5 phenotype (Table 2, 3), rarely seen in subtype B infected adult patients. In addition, the chimeras from the patients with advanced disease status (III, IV) replicated more efficiently than the less advanced disease patients (I, II) [Table 1, Figs. 3 and 4]. Furthermore, it has also been found that the percentage of CD4 T cells expressing CCR5 in Indian adults is higher than among Caucasian races [39]. It is, therefore, likely that due to the presence of this larger pool of CCR5 positive CD4 cells, the virus may not need a coreceptor switch during disease progression.

Our data showed that subtype C V3–V5 region chimeras replicated better in primary blood T-lymphocytes (Fig. 3) and MDM (Fig. 4) compared with subtype B chimeras, HIV-1_NL4-3 _and subtype B primary isolates. In general, there was a correlation between higher virus entry, earlier replication peaks, and increased replication efficiencies in primary T-lymphocytes and MDM. Examination of sequence data published earlier on these patients [5] and presented in Fig. 1 and Table show several features of subtype C chimeras, including number of positive charges, amino acid sequence variation and number of glycosylation sites. One striking feature was that the chimeras from patient AP.17 (chimeras # 171, 173) and AP.5 (chimeras # 512, 514) retained the N-linked glycosylation site that includes the first cysteine residue of V3 loop [10], whereas other chimeras had mutations in this site, contributing to the differences in replication efficiencies of these chimeras (Fig. 3). Our subtype V3–V5 region chimeras were in close resemblance with subtype C (HIV-1C) with V3 sequences similar to HIV-1_BAL _(R5), as shown in Fig. 1. The motif SIHIGPGRALYTTGEIIGDI that is important for R5 tropism as seen for HIV-1_BAL _in Fig. 1 was fairly conserved in our subtype C chimeras contributing to R5 tropism. Similarly subtype C chimeras V4–V5 region sequences show similarity to subtype C sequence but variability to subtype B (X4 and R5) sequences. The difference in amino acid sequences in V3 to V5 regions in subtype C chimeras as compared to subtype B sequences may be responsible for increased replication efficiencies of subtype C chimeras. Further studies on site directed mutagenesis of the V3–V5 regions and binding affinity of gp120 to CCR5 and/or gp120 incorporation into chimeric virions might pinpoint the major difference in replication efficiencies.

While a co-infection *in vitro *study with more fit subtype B and less fit subtype C viruses indicates more fitness of subtype B over subtype C viruses [40], several *in-vivo *infection studies on rhesus macaques have shown that HIV-1 subtype C *env *chimeric viruses demonstrate greatly enhanced infectivity [41-43] and replication efficiency as compared to subtype B and E viruses [44,45]. Our data on higher levels of HIV-1 entry and increased replication efficiencies of subtype C chimeras compared with subtype B viruses is consistent with the latter *in vivo *studies [41-43]. Increased replication efficiency of subtype C viruses has also been attributed to the presence of an extra NF-κB site in the LTR of subtype C viruses [46,47]. However, these viruses have shown not only to replicate efficiently but to also be transmitted and spread more efficiently than other HIV-1 subtypes [1,9]. During horizontal and vertical transmission subtype C viruses have been shown to spread more rapidly than other subtypes due to increased mucosal and vaginal shedding [36,48,49]. This increased transmission cannot solely be explained due to increased LTR activity as increased shedding of virus and increased transmission may be attributed to the envelope gene and others regions and functions of the virus.

While various HIV-1 subtypes prevalent in different regions of the world show variability in their replication kinetics and disease progression in infected individuals [32], HIV-1 subtype C is rapidly spreading and has already become the predominant subtype worldwide. The data presented in this paper indicate that HIV-1 envelope V3–V5 region of subtype C contributes to increased rates of replication as compared to subtype B. This would also explain higher viral loads and faster disease progression in patients infected with HIV-1 subtype C [35] as well as increased shedding of virus seen in subtype C infected population [36,48]. Our data shows the HIV-1 subtype C envelope V3–V5 region may be one of the determinants of increased virus entry and replication. These results provide another candidate gene to be responsible for the rapid spread of HIV-1 subtype C and its ability to dominate in populations that initially had higher incidence of other subtypes.

## Methods

### Construction of HIV-1 Chimeras

Sixteen previously characterized envelope V3–V5 region sequences (Fig. 1) from nine subtype C Indian isolates (Table 1) [5] were reciprocally substituted into pNL4-3 in *Bgl*II-*Bgl*II sites flanking the region. The 650 bp fragment encompassing the V3–V5 region corresponds to amino acids 240–439 of gp120 of the HIV-I_NL4-3 _sequence with the V3-loop positioned between amino acids 267 and 300. PCR primers were designed to amplify the V3–V5 region and to replace a similar region in pNL4-3 by engineering a *Bgl*II site at the 3' end at position 7611 corresponding to all isolates. PCR amplification was carried out using Pfu polymerase. The ~600 bp PCR product was digested with *Bgl*II and cloned into pGEM (NL4-3) using the *Bgl*II sites [50]. The *Bgl*II-*Bgl*II fragment was reciprocally exchanged into the pGEM plasmid containing the *Eco*RI-*Bam*HI fragment as there were no *Eco*RI or *Bam*HI sites in this a region in any Indian isolate except AP.5 which contained an *Eco*RI site in the sequence. The recombinant clones were checked by digestion with *Bgl*II and the orientation was confirmed by DNA sequencing using primer (5'TCAACTGCTGTTAAATGGC3'). Finally, the modified *Eco*RI-*Bam*HI fragment containing the sporadic subtype C Indian isolate V3 to V5 region was reciprocally substituted into pNL4-3. Two clones were obtained from each patient sample and these are numbered with numerals of patient identification number followed by clone number. All the clones were again checked by digestion with *Eco*RI/*Sal*I and *Bam*HI and confirmed by sequencing.

### Cell lines and primary cells

HeLa cells, U373-MAGI-CXCR4 and U373-MAGI-CCR5 cell lines were cultured in DMEM with 10% fetal bovine serum (FBS) and penicillin-streptomycin (Invitrogen). The MAGI cell media was also supplemented with 0.2 mg/ml of G418, 0.1 mg/ml of hygromycin B and 1 μg/ml of puromycin. T-lymphocyte cell lines, A3.01 and MT-2 were cultured in RPMI 1640 supplemented with 10% FBS and penicillin-streptomycin. Peripheral blood mononuclear cells (PBMC) were obtained by single step density gradient centrifugation with Ficoll-Hypaque (Amersham) from whole blood of normal donors. The blood collections were made after informed consent, and were approved by the Human Subject Ethical committees of International Center for Genetic Engineering and Biotechnology (ICGEB) and the Human Subjects Committee of the University of Arizona (Tucson, AZ) and were based on Indian Council of Medical Research (ICMR) and National Institutes of Health (NIH) guidelines, respectively. PBMC were collected, washed twice with cold PBS and centrifuged at 1000 rpm for 10 min to avoid collecting platelets. Primary monocyte/macrophages and peripheral blood lymphocytes (PBL) were separated from PBMC using magnetic bead isolation protocols. Monocytes were isolated by CD14 magnetic bead isolation (Miltenyi biotech) according to the manufacturer's protocols. The cells bound to the CD14 bead were used as primary monocytes. The primary monocytes eluted from the CD14 isolation columns were counted and plated in 48 well plates at 1 × 10^6 ^cells/well in RPMI 1640 with 15% human AB serum (Gemini biotech) and MCSF (Sigma) for 7 days and were allowed to differentiate into macrophages in this media. The cells were fed every two days during differentiation. The cells collected as unbound flow through from the CD14 bead isolation protocol were used as PBL. PBL were cultured in RPMI 1640 with 10% FBS and penicillin-streptomycin. PBL were stimulated with 5 μg/ml of PHA for 24–48 h. The stimulated cells were washed with PBS and resuspended in RPMI 1640 with 10% FBS and 20 U/ml of recombinant human IL-2 (Invitrogen).

### DNA Transfections

Subtype C chimeric proviral DNAs were transfected in HeLa cells by electroporation as described before [34] or by Lipofectamine 2000 (Invitrogen) [51]. For the Lipofectamine method, HeLa cells were grown in DMEM with 10% FBS and penicillin-streptomycin to about 80% confluency. The cells were then split and counted and plated in a 6-well plate at 10^5 ^cells/well in DMEM with 10% FBS without antibiotics. The cells were transfected the next day with 3 μg DNA in DNA-lipofectamine complexes as per manufacturer's procedure. Chimeric viruses were harvested by collecting culture supernatant from the wells 72 hrs post-transfections. Virus production was measured by a reverse transcriptase (RT) assay [34,51].

### Infections

A3.01 cells (2 × 10^6^), MT-2 (2 × 10^6^), PBL (1 × 10^6^) and MDM (0.5 × 10^6^) per well were cultured and infected with equal amounts of subtype C *env *region chimeras, subtype B V3 region chimeras (M5g, M7f, M1c) [34,50], subtype B and C primary isolates, and virus production was measured every 3 days. We used two subtype C (3041-R5 and 5441-X4) and two subtype B (2099-R5 and 2101-X4/R5) primary isolates that were obtained from AIDS Research and Reference Reagent Program as controls. Briefly, viruses were adsorbed in A3.01 cells, MT-2 and PBL for 90 min in serum free media at 37°C and 5% CO_2_. After incubation, 500 μl of appropriate media containing serum and antibiotics were added. MDM were infected in media containing serum and polybrene (8 μg/ml) incubated at 37°C and 5% CO_2 _for 16 hrs. After incubation, the macrophages were washed in PBS to remove polybrene and resuspended in macrophage culture media.

### Coreceptor utilization

U373-MAGI-CXCR4 and U373-MAGI-CCR5 cell lines were plated at ~6 × 10^4 ^cells/well in 24 well plate in complete DMEM with G418-hygromycin-puromycin. Both cells lines were infected with 5,000 and 10,000 cpm (RT assay counts) of chimeric subtype C virus and primary isolate controls diluted in a total volume of 300 μl of complete DMEM (without antibiotics) with DEAE-dextran (final concentration 20 μg/ml). Two hours post-adsorbtion, 1.5 ml of fresh MAGI media was added. To assess the rate of entry, 40 h post-infection, the medium was removed and cells were fixed in 1% formaldehyde and 0.2% glutaraldehyde for 5 min. Then, the cells were washed with PBS and stained for 2 hrs in staining solution containing 0.2 M potassium ferricyanide, 0.2 M potassium ferrocyanide, 2 M MgCl_2 _and 40 μg/ml X-Gal. After staining, the cells were washed in PBS and resuspended in PBS with Sodium Azide. Cells stained blue were counted immediately and counts were estimated as number of blue cells per well of infected cells.

### Cytopathic effects (MT-2 assay)

The syncytium-inducing ability of the subtype C envelope chimeras was determined and compared to a known syncytium-inducing (SI) virus (HIV-1_NL4-3_) and a non-syncytium-inducing (NSI) viruses (HIV-1_BaL _and HIV-1_Ada-M_) by infecting the MT-2 cell line with equal amounts of these viruses. Syncytium formation was monitored every day in the cultures up to 30 days post-infection. The viruses that formed more than four syncytia per field of view in the culture were designated as SI viruses (ACTG protocol), whereas viruses that failed to induce this number of syncytia in the culture were termed NSI viruses.

## Competing interests

The author(s) declare that they have no competing interests.

## Authors' contributions

VS and SRD contributed equally to this work. SRD carried out the PCR, cloning, and sequencing. VS performed all biological experiments, including T-cells lines and primary cells infection experiments. RR contributed in transfections and infection experiments. SS and SG contributed to the collection of patients' samples and clinical data. VS, SRD, RR, NA and SJ participated in the experimental design, data interpretation and writing of the manuscript. All the authors read and approved the final manuscript.
